# Ethical issues with psychedelic-assisted treatments in psychiatry: A systematic scoping review

**DOI:** 10.1017/S0033291725101761

**Published:** 2025-09-29

**Authors:** Chiara Caporuscio, Christopher Poppe, Astrid Gieselmann, Dimitris Repantis

**Affiliations:** 1 Charité – Universitätsmedizin Berlin, corporate member of Freie Universität Berlin and Humboldt-Universität zu Berlin, Department of Psychiatry and Neurosciences, Campus Charité Mitte, Berlin, Germany; 2 Centre for Philosophy and AI Research PAIR, Friedrich-Alexander-Universität, Erlangen-Nürnberg, Germany; 3 German Center for Mental Health (DZPG), Partner Site Berlin-Potsdam, Germany; 4 Charité – Universitätsmedizin Berlin, corporate member of Freie Universität Berlin and Humboldt-Universität zu Berlin, Department of Psychiatry and Neurosciences, Campus Benjamin Franklin, Berlin, Germany

**Keywords:** ethics, psychedelic-assisted treatments, psychiatry, scoping review

## Abstract

Based on promising preliminary results from clinical trials, it seems likely that psychedelic substances (classic serotonergic psychedelics, such as psilocybin, and entactogens, such as MDMA) will be introduced into psychiatry as psychedelic-assisted therapy. This also raises a range of ethical questions that urgently need to be addressed before widespread roll-out in society. This scoping review fills a gap in the literature by providing an overview of these ethical issues using a systematic search, presentation, and descriptive analysis of ethical issues in psychedelic-assisted treatments. It includes peer-reviewed studies pertaining to human study participants and psychiatric patients (population), which discuss ethical issues (concept) of psychedelic treatments (context) in clinical trials and other clinical applications. The systematic search included several databases: MEDLINE, PsycInfo, CINAHL, HeinOnline, and PsycArticles. The search strategy, including all identified keywords and index terms, was adapted for each included database. The search was completed in June 2025 and studies published until then in any language were included. After an iterative process of inductive and deductive coding of ethical issues, the scoping review comprises seven themes related to the ethics of psychedelic-assisted treatments: (1) safety and patient well-being, (2) therapeutic relationships, (3) informed consent, (4) equity and access, (5) research ethics, (6) special contexts, and (7) societal and cultural implications. The results can be used to inform and stimulate further discussion and in-depth research on the ethics of psychedelic-assisted treatments, possibly leading to more nuanced debate surrounding a safer and more ethical implementation of psychedelic-assisted treatments in the future.

## Introduction

Psychedelics constitute a distinct category of pharmacological interventions that induce alterations in sensory perception, cognition, and one’s sense of self. This class encompasses serotonin 2a receptor agonists like lysergic acid diethylamide (LSD), mescaline, psilocybin, and dimethyltryptamine (DMT). The term is sometimes used in a broader sense to include glutamate antagonists such as ketamine and phencyclidine, along with entactogens like 3,4-methylenedioxymethamphetamine (MDMA). Despite their therapeutic use in the 1950s and 1960s, research on psychedelics faced severe restrictions due to strict scheduling in subsequent decades. Most psychedelics have been classified as controlled substances with no recognized medical applications, nonetheless underground use for mental health treatment has persisted. Interest in psychedelics has revived since the 1990s, with emerging research suggesting their efficacy in treating conditions like depression, addiction, posttraumatic stress disorder (PTSD), and end-of-life anxiety. Clinical trials of psychedelic-assisted treatments are increasingly conducted, and recent limited access programs, for example, in Australia for psilocybin and MDMA to treat resistant depression and PTSD, highlight their potential for therapeutic use.

The use of psychedelics presents several ethical challenges. Psychedelic experiences can lead to profound changes in beliefs and personality, making the predictability and validity of informed consent questionable. Furthermore, the altered states of consciousness induced by these substances can increase susceptibility to abuse and misconduct. Incorrect dosing, inadequate guidance, or an unsuitable context can lead to challenging experiences, and psychedelics have been associated with a risk for psychotic symptoms, especially in individuals with a family history of psychosis (Sabé et al., 2025; Simonsson, Johnson, & Hendricks, 2021). Research so far has focused on specific ethical issues, such as abuse in the patient-therapist relationship (McNamee, Nese, & Buisson, 2023) or informed consent (Jacobs, 2023). A narrative review by Schlag, Aday, Salam, Neill, and Nutt (2022) critically examines the evidence for potential harms of psychedelics, such as abuse liability, potential for dependence, toxicity, and overdose and concludes that most of these risks are minimal or unsupported by the available evidence. However, ethical issues of psychedelic-assisted treatments encompass more than potential physical harms of psychedelic substances. As the use of psychedelics in clinical settings remains relatively novel, concerns about social and cultural issues, patient safety and autonomy, and power abuse are raised among stakeholders, regulatory agencies, and the general public. The lack of a well-established ethical framework for integrating psychedelic-assisted treatments into mainstream medicine poses a serious obstacle to the responsible medicalization of these substances. It is therefore crucial to systematically examine the landscape of ethical issues relevant to psychedelic-assisted treatments in order to further understanding in this field.

A comprehensive search of MEDLINE, the Cochrane Database of Systematic Reviews, Open Science Framework, and PROSPERO revealed no existing or ongoing systematic or scoping reviews on this topic. Consequently, a cohesive overview of the ethical challenges inherent to psychedelic-assisted treatments is warranted. This scoping review aims to fill this gap by systematically mapping the research published before June 2025 on the ethics of psychedelic-assisted treatments. Specifically, this study focuses on legal contexts, namely, clinical trials and regulated psychotherapeutic or psychiatric practice. Unregulated use is excluded to focus the review on the most relevant academic research informing regulatory and reclassification decisions. By focusing on regulated use, this scoping review aims to clarify and assess the ethical implications of psychedelic-assisted treatment for patients, therapists, healthcare providers, policymakers, and society at large.

## Methods

### Preregistration

The protocol for this scoping review was preregistered on Open Science Framework on February 19, 2024.

### Search Strategy and selection criteria

We conducted a PRISMA-compliant scoping review following the frameworks outlined by the JBI Manual of Evidence Synthesis on scoping reviews (Peters et al., 2020) and the Preferred Reporting Items for Systematic Reviews and Meta-Analyses Extension for Scoping Reviews (PRISMA-ScR) Checklist (Tricco et al., 2018). This scoping review provides the full spectrum of ethical issues to navigate this complex landscape that intersects medical, legal, and philosophical domains (Strech & Sofaer, 2012).

### Eligibility criteria

We included a paper if:

1. The paper discussed ethical issues of the use of psychedelics in psychiatry or social and legal challenges of the use of psychedelics in psychiatry.
2. The paper reported on psychedelic (classic psychedelics, entactogens, and ketamine) treatments in clinical trials or other clinical applications.
3. The paper was peer reviewed.

We excluded a paper if:

1. The paper was not from an academic source, such as gray literature, books, or blog posts.

### Information sources

The following databases were initially searched from their inception until February 2, 2024: MEDLINE, PsycInfo, CINAHL, HeinOnline, and PsycArticles. Furthermore, we tracked citations and references of the included articles from the database search using Citation Chaser (https://estech.shinyapps.io/citationchaser/). A second search on MEDLINE via OVID and PubMed included articles published between February 2024 and June 20, 2025.

### Search strategy

The search strategy incorporated variations of the terms ‘psychiatry’, ‘ethics’, and ‘psychedelics’. The searches were tailored to align with the capabilities of each database. Databases were searched across all available dates and publication types. The searches were cross-checked by the team to ensure reproducibility. The full electronic search is included in the Supplementary Material.

### Study selection

The papers identified through the electronic search were compiled into a Zotero database, and duplicates were removed. Titles and abstracts were independently screened for relevance by two team members (C.C. and C.P.). Any disagreements were resolved through discussion until a consensus was reached. The full texts were screened following the same process, with any disagreements resolved through consultation with a third team member (A.G.).

### Data analysis

**Figure 1.**
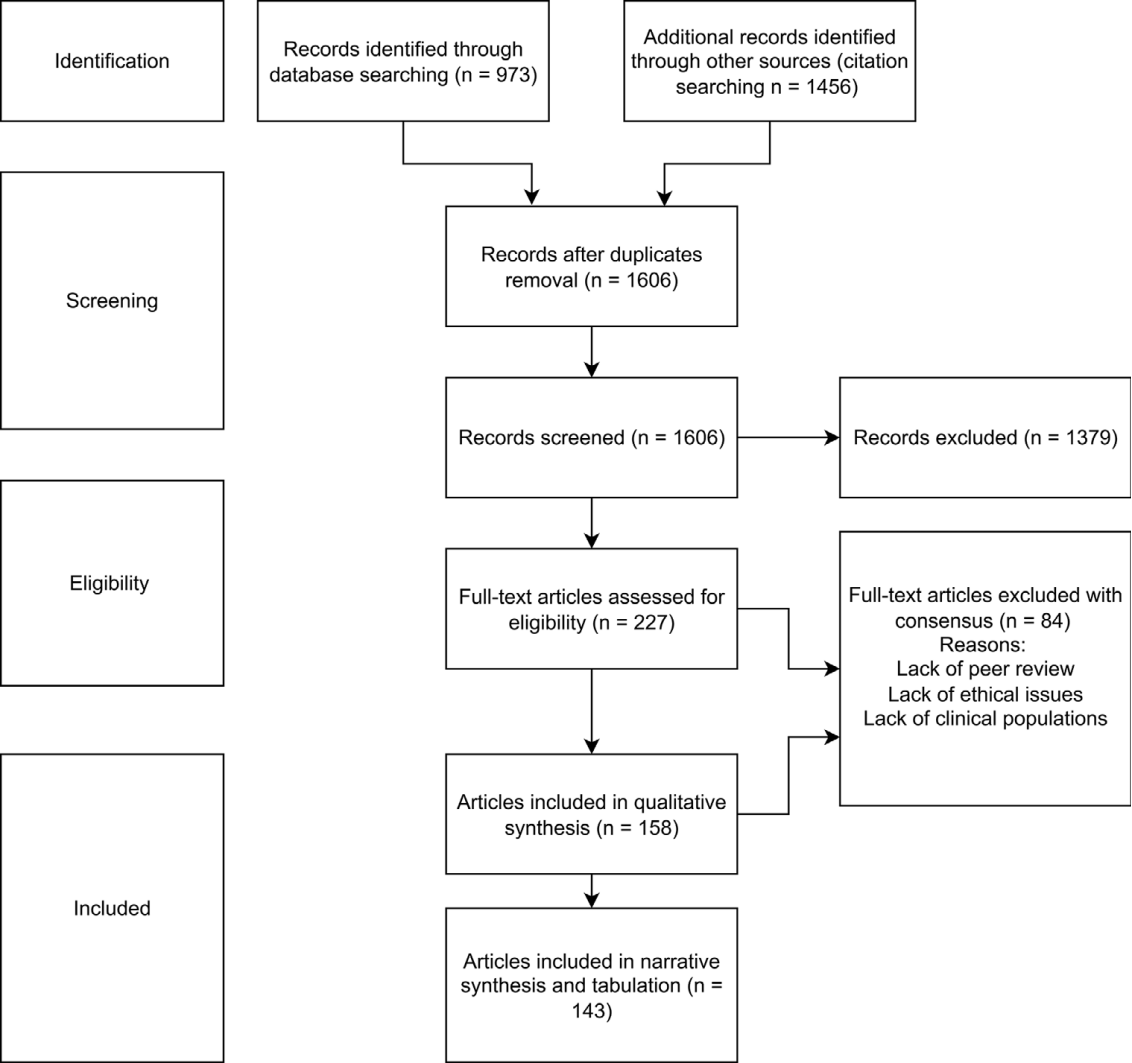
PRISMA.

The selected papers were imported into the coding software MAXQDA (VERBI, 2024) and thematic analysis, adapted from Braun and Clarke (2006), was employed to synthesize the key reasons. An initial coding framework was developed after thoroughly reviewing all the articles (A.G., C.C., and C.P.). All articles were analyzed by either C.C., C.P., or A.G. Disagreements regarding coding were resolved through discussions among these three researchers until consensus was reached. All ethical arguments were coded, regardless of whether they were endorsed by the authors of the article. An inductive process was employed to iteratively refine and expand the initial coding framework and themes until all articles were thoroughly analyzed. The final themes and subthemes were then discussed with the entire research team and further refined to achieve consensus (Figure 1).

## Results

**Figure 2.**
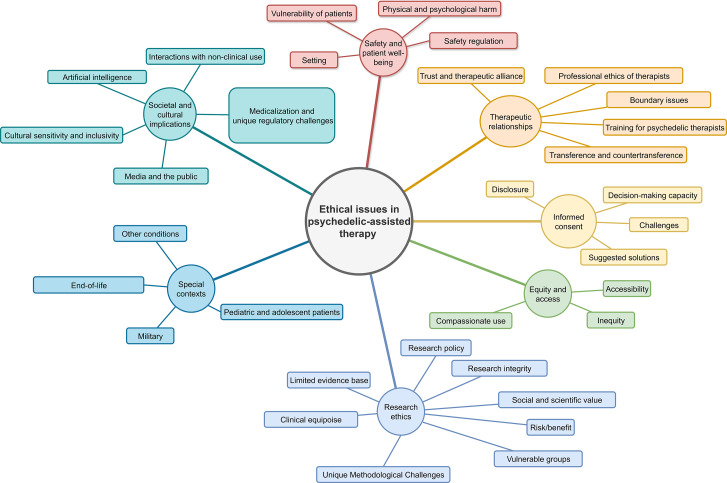
Visualization of themes and subthemes.

The analysis resulted in seven themes: (1) safety and patient well-being, (2) therapeutic relationships, (3) informed consent, (4) equity and access, (5) research ethics, (6) special contexts, and (7) societal and cultural implications. These themes were made of subthemes, in which codes were grouped together (Figure 2). An overview of themes, subthemes, and the referencing articles can be found in Table 1.

**Table 1.** Themes after qualitative coding: a publication can be included in more than one subtheme/theme if it included multiple arguments

Themes	Subthemes	Articles mentioning the themes:
Safety and patient well-being	Vulnerability of patients Physical and psychological harm Safety regulation Setting	Anderson, Danforth, & Grob, 2020; Azevedo, Miguel, & Madeira, 2023; Villiger & Trachsel, 2023; Johnson, 2021; Seybert et al., 2023; Greif & Šurkala, 2020; Edelsohn & Sisti, 2023; Smith & Sisti, 2021; Romero, 2023; Harrison, Faber, Zare, Fontaine, & Williams, 2025; Palitsky et al., 2024; Simonsson, Johnson, & Hendricks, 2024; Poppe & Repantis, 2024; Repantis, Koslowski, & Fink, 2024; Sjöstedt-Hughes, 2023; Strauss et al., 2022; Holoyda, 2023; Scala et al., 2024; Sisti, Segal, & Thase, 2014; Letheby & Saja, 2022; Yaden, Earp, & Griffiths, 2022; Cheung, Earp, & Yaden, 2023; Brennan & Belser, 2022; Villiger, 2024a, 2024b; Wang, Mathai, Gukasyan, Nayak, & Garcia-Romeu, 2024; Williams, Card, & Faber, 2023; Zhang, Wang, Gao, & Wang, 2024. Smith & Sisti, 2021; Zhang & Ho, 2017; Jacobs, 2023; Noorani, 2020; Rea & Wallace, 2021; Schlag et al., 2022; Johnson, Richards, & Griffiths, 2008; Johnson, Griffiths, Hendricks, & Henningfield, 2018; McNamee et al., 2023; Ryan & Loo, 2017; Sandbrink et al., 2024; Barnes, 1970; Lee, Rosenbaum, & Buchman, 2024. Barber & Dike, 2023; Belouin et al., 2022; Cheung et al., 2023; Yaden et al., 2022; Villiger, 2024a, 2024b; Zhang et al., 2024. Barber & Dike, 2023; Barnes, 1970; Villiger, 2024a, 2024b; Barber, Nemeroff, & Siegel, 2022; Schenberg & Gerber, 2022; Jacobs, 2023; Jacobs, Murphy-Beiner, Rouiller, Nutt, & Spriggs, 2024.
Therapeutic relationships	Trust and therapeutic alliance Professional ethics of therapists Boundary issues Transference and countertransference Training for psychedelic therapists	Ortiz et al., 2022; Barnes, 1970. Brennan & Belser, 2022; Jacobs, 2023; Whinkin, Opalka, Watters, Jaffe, & Aggarwal, 2023; Rajwani, 2023; Azevedo et al., 2023; Harrison et al., 2025; Herpers, Maximets, van Dongen, Zijlmans, & Vermetten, 2024; Jacobs et al., 2024; Neitzke-Spruill, Devenot, Sisti, Averill, & McGuire, 2024; O’Donnell, Grigsby, & Grob, 2025; Cohen & Marks, 2025; Borkel et al., 2025; Poppe, Villiger, Repantis, & Trachsel, 2025; Caporuscio & Fink, 2024; Timmermann, Watts, & Dupuis, 2022. Holka-Pokorska, 2023; Johnson, 2021; McNamee et al., 2023; Lee et al., 2024; Peacock et al., 2024; Phelps, 2017; Repantis et al., 2024; Smith & Sisti, 2021; Harrison et al., 2025; Poppe & Repantis, 2024; Sisti, 2024; Herpers et al., 2024; Jacobs, Earp, et al., 2024; Neitzke-Spruill, Beit, Averill, & McGuire, 2025, McGuire et al., 2024. Phelps, 2017; Anderson et al., 2020; Marcus, 2023; McNamee et al., 2023; Jacobs et al., 2023; Villiger & Trachsel, 2023. Anderson et al., 2020; Azevedo et al., 2023; Barber & Dike, 2023; Barber et al., 2022; Barnes, 1970; Barnett & Greer, 2021; Belouin et al., 2022; Brennan & Belser, 2022; Destoop et al., 2025; Emmerich & Humphries, 2023; Holka-Pokorska, 2023; Hall, 2021; King & Hammond, 2021; Pilecki, Luoma, Bathje, Rhea, & Narloch, 2021; Perez Rosal et al., 2024; Poppe & Repantis, 2024; Poppe et al., 2025; Siegel et al., 2023; Sisti, 2024; Smith, Faber, Buchanan, Foster, & Green, 2022; Thal et al., 2022; Williams et al., 2023; Wilson-Poe et al., 2024.
Informed consent	Disclosure Decision-making capacity Challenges Suggested solutions	Barnes, 1970; Edelsohn & Sisti, 2023; Mathai et al., 2022; Ortiz et al., 2022; Villiger, 2024a, 2024b; Repantis et al., 2024; Pilecki et al., 2021; Barber & Dike, 2023; Anderson et al., 2020; Egerton & Capitelli-McMahon, 2023; Emmerich & Humphries, 2023; Haeusermann & Chiong, 2024; Jacobs, 2023; Jacobs, 2023; Jacobs, Earp, et al., 2024. Haeusermann & Chiong, 2024; McNamee et al., 2023; Sisti et al., 2014; Villiger, 2024a, 2024b; Lee et al., 2024; Otterman, 2023; Peterson, Largent, Lynch, Karlawish, & Sisti, 2023; Cheung et al., 2023. Haeusermann & Chiong, 2024; Jacobs, 2023; Letheby & Saja, 2022; Mathai et al., 2022; Otterman, 2023; Repantis et al., 2024; Smith & Appelbaum, 2022; Smith & Sisti, 2021; Villiger, 2024a, 2024b; da Costa & Sofuoglu, 2023; Anderson et al., 2020; Barnes, 1970; Barnett & Greer, 2021; Azevedo et al., 2023; Barber & Dike, 2023; Elman, Pustilnik, & Borsook, 2022; O′ Brien & Nutt, 2025; Poppe & Repantis, 2024; McGuire et al., 2024. Villiger, 2024a, 2024b; Haeusermann & Chiong, 2024; Jacobs, 2023; Lee et al., 2024; Letheby & Saja, 2022; Rosenbaum, Cho, Schneider, Hales, & Buchman, 2023; Seybert et al., 2023; Smith & Sisti, 2021; Villiger & Trachsel, 2023; Barnett & Greer, 2021; Bodnár & Kakuk, 2019; Tustison, Niemi, & Choi, 2025; Destoop et al., 2025; Cohen & Marks, 2025; Poppe & Repantis, 2024; Marks, Brendel, Shachar, & Cohen, 2024.
Equity and access	Inequity Accessibility Compassionate use	Barber & Dike, 2023; Barnett & Greer, 2021; Black, 2023; Cheung et al., 2023; Jacobs, 2023; King & Hammond, 2021; Lee et al., 2024; Manson et al., 2023; Michaels, Purdon, Collins, & Williams, 2018; Mintz et al., 2022; Munafò et al., 2023; Noorani, 2020; Ona et al., 2022; Peterson et al., 2023; Pilecki et al., 2021; Rosa, 2022; Rudolph, 2023; Sisti et al., 2014; Smith & Appelbaum, 2022; Wolfgang & Hoge, 2023; Poppe & Repantis, 2024; Jacobs, Earp, et al., 2024; Bartlett, Christ, Martins, Saxberg, & Ching, 2024. Ortiz et al., 2022; Barber et al., 2022; González Romero, 2023; Barber & Dike, 2023; Barnett & Greer, 2021; Belouin et al., 2022; Campbell & Williams, 2021; Haeusermann & Chiong, 2024; Lee et al., 2024; Miceli McMillan, 2022; Noorani, 2020; Ona et al., 2022; Rea & Wallace, 2021; Rosa, 2022; Smith & Appelbaum, 2022, Smith et al., 2022; Strauss et al., 2022; Whinkin et al., 2023; Williams et al., 2023, Berens & Kim, 2022; Cheung et al., 2023; Hall, 2021; Siegel, Daily, Perry, & Nicol, 2023; Zhang et al., 2016; Zhang & Ho, 2017; McMillan, 2021; Williams, Cabral & Faber, 2023; Zhang et al., 2016; Zhang & Ho, 2017; McMillan, 2021; Poppe & Repantis, 2024; Perez Rosal et al., 2024; Wolfson & Valid, 2024; Bayrhammer-Savel, Ortner, Van Hout, & Komorowski, 2024; Tustison et al., 2025; Destoop et al., 2025. Campbell & Williams, 2021; Greif & Šurkala, 2020; Hall, 2021.
Research ethics	Research policy Research integrity Social and scientific value Risk/benefit Vulnerable groups Unique methodological challenges Clinical equipoise Limited evidence base	Ona et al., 2022; Hall, 2021; Andreae et al., 2016; Belouin et al., 2022; Campbell & Williams, 2021; Haeusermann & Chiong, 2024; Poppe & Repantis, 2024. Andreae et al., 2016; da Costa & Sofuoglu, 2023; Bodnár & Kakuk, 2019; Oliveira-Maia & Seybert, 2024; Jacobs, Earp, et al., 2024; Tang, 2024. da Costa & Sofuoglu, 2023; Bodnár & Kakuk, 2019; Peterson, Timmermann, & Weijer, 2019. da Costa & Sofuoglu, 2023; Bodnár & Kakuk, 2019; Andreae et al., 2016; Jacobs, Earp, et al., 2024; Tang, 2024; O’Brien & Nutt, 2025. da Costa & Sofuoglu, 2023; Bodnár & Kakuk, 2019; Andreae et al., 2016; Azevedo et al., 2023; Rudolph, 2023; Anderson et al., 2020; Barber & Dike, 2023; Elman et al., 2022; George, Michaels, Sevelius, & Williams, 2020; Simon, 2024; Smith et al., 2022; Black, 2023; Edelsohn & Sisti, 2023; Strauss et al., 2022; Williams et al., 2023; Zhang & Ho, 2017; Bartlett et al., 2024. Anderson et al., 2020; Andreae et al., 2016; Azevedo et al., 2023; Barber & Dike, 2023; Beswerchij & Sisti, 2022; Bodnár & Kakuk, 2019; Elman et al., 2022; Haeusermann & Chiong, 2024; Iserson, 2019; Kious, Schwartz, & Lewis, 2023; Ona et al., 2022; Rucker & Young, 2021; Sandbrink et al., 2024; Videira et al., 2024; Villiger, 2024a, 2024b; Villiger & Trachsel, 2023; Zhang & Ho, 2017; da Costa & Sofuoglu, 2023; Destoop et al., 2025; Matvey et al., 2025; Oliveira-Maia & Seybert, 2024; Tang, 2024; Scala et al., 2024. Barber et al., 2022; Barber & Dike, 2023; Poppe & Repantis, 2024. Barber & Dike, 2023; Munafò et al., 2023; Ona et al., 2022; Destoop et al., 2025; Jacobs, Earp, et al., 2024.
Special contexts	Pediatric and adolescent patients Military End of life Other conditions	Edelsohn & Sisti, 2023; Sutherland, Ho, & Croarkin, 2025; Jeffrey, Weintraub, & Grob, 2024; Sathappan & Yudkoff, 2024; Otterman, 2023. Germann, 2019; Hoener, Wolfgang, Nissan, & Howe, 2023; Wolfgang & Hoge, 2023. Berens & Kim, 2022; Rosenbaum, Cho, et al., 2023; Somers & Scheepers, 2025; Cornish et al., 2025; Poppe & Repantis, 2024; Korkmaz, Cikrikcili, Akan, & Yucesan, 2024; Masse-Grenier et al., 2024; Plourde et al., 2024. Peterson et al., 2019; Peterson et al., 2023; Rudolph, 2023; Cardone et al., 2024; Zheng, Ma, Yang, & Li, 2024; Hu, Lin, Wang, & Wang, 2025; Otterman, 2023; Lacroix, Fatur, Hay, Touyz, & Keshen, 2024; Robinson et al., 2024.
Societal and cultural implications	Interactions with non-clinical uses Medicalization and unique regulatory challenges Cultural sensitivity and inclusivity Media and the public Artificial intelligence	Barber & Dike, 2023; Letheby & Saja, 2022; Williams et al., 2023; Andrews, Hall, Humphreys, & Marsden, 2025. Miceli McMillan, 2021, 2022; Noorani, 2020; Ona et al., 2022; Andrews & Wright, 2022; Belouin et al., 2022; Celidwen et al., 2022; Gerber et al., 2021; Rea & Wallace, 2021; Rudolph, 2023; Whinkin et al., 2023; Destoop et al., 2025, Poppe & Repantis, 2024; Jacobs, Earp, et al., 2024; Barnes, 1970; Miceli McMillan, 2021; Azevedo et al., 2023; Hall, 2021; Noorani, 2020; Belouin et al., 2022; Barber & Dike, 2023. Azevedo et al., 2023; Barber & Dike, 2023; Lee et al., 2024; Rea & Wallace, 2021; Belouin et al., 2022; Barber et al., 2022; Celidwen et al., 2022; King & Hammond, 2021; Miceli McMillan, 2021, 2022; Ona et al., 2022; Peacock et al., 2024; Rea & Wallace, 2021; Rosa, 2022; Hauskeller & Schwarz, 2023; Marcus, 2023; Labate, de Assis, Gomes, Smith, & Cavnar, 2022; Schenberg & Gerber, 2022; Gerber et al., 2021; Haeusermann & Chiong, 2024; Williams et al., 2023; Jacobs, Earp, et al., 2024; O’Donnell et al., 2025; Cohen & Marks, 2025; McGuire et al., 2024; Neitzke-Spruill et al., 2024. Campbell & Williams, 2021; Barber et al., 2022; Azevedo et al., 2023; Pilecki et al., 2021; Ryan & Loo, 2017; Sandbrink et al., 2024; Munafò et al., 2023; Noorani, 2020; Ona et al., 2022; Peterson et al., 2023; Belouin et al., 2022; Beswerchij & Sisti, 2022; Cohen & Marks, 2025; O’Brien & Nutt, 2025; Sisti, 2024; Cheung, Earp, Patch, & Yaden, 2025. Sarris, Halman, Urokohara, Lehrner, & Perkins, 2024.

### Safety and patient well-being

Ensuring the safety and well-being of patients in psychedelic therapy is a fundamental ethical concern. Our analysis identified four related subthemes: (1) vulnerability of patients, (2) physical and psychological harm, (3) safety regulation, and (4) setting. Patient vulnerability emerged as the key ethical concept, while the other four subthemes represent contextual factors that impact the risk of harm. In psychedelic therapy, the subjective effects of psychedelics make patients particularly vulnerable to emotional distress, undue influence, inducement, sexual abuse, suggestibility, and various forms of exploitation (Anderson et al., 2020; Azevedo et al., 2023; Barber et al., 2022; Barber & Dike, 2023; Barnes, 1970; Barnett & Greer, 2021; Belouin et al., 2022; Brennan & Belser, 2022; Hall, 2021; Harrison et al., 2025; Holka-Pokorska, 2023; Siegel et al., 2023; Simonsson et al., 2024; Thal et al., 2022; Wang et al., 2024; Williams et al., 2023).

Vulnerable patients might experience both physical harm and psychological harm. Assessment of adverse events has been lagging, and new frameworks are being created to identify risks and harms (Palitsky et al., 2024). While the risks of dependence, abuse and neurotoxicity seem to be extremely low, especially for classic psychedelics like LSD or psilocybin (Johnson et al., 2008; Schlag et al., 2022), there are cases of psychedelics triggering hallucinogen persisting perception disorder or psychosis in predisposed subjects (Scala et al., 2024; Schlag et al., 2022). For this reason, there has been research for development of ‘non-hallucinogenic psychedelics’, in order to elicit many of the neurobiological changes without the subjective effects. Some ethical issues emerge from this discussion, for example, about whether psychedelics should be the default option if non-hallucinogenic psychedelics were an option (Cheung et al., 2023; Poppe & Repantis, 2024; Yaden et al., 2022; Zhang et al., 2024).

In order to mitigate the risks of psychedelics, the safety of both the therapeutic setting and the substances themselves is crucial. Given the powerful nature of psychedelic experiences, there is a need for clear safety measures and strict oversight regarding therapeutic environments, ensuring they are safe, supportive, and conducive to treatment (Villiger, 2024a, 2024b; Zhang et al., 2024). Controlled settings must include proper screenings, preparation, and support, and are therefore deemed safer compared to unregulated settings such as underground therapies, retreats or ceremonial use (Barber et al., 2022; Jacobs, Murphy-Beiner, et al., 2023; Villiger, 2024a, 2024b). This is particularly problematic because of the risk that trial participants might attempt to replicate the experience after the trial ends (Jacobs, Murphy-Beiner, et al., 2023). However, some scholars recognize the value of a ritualistic context compared to a clinical setting, especially for substances with a long history of traditional use (Schenberg & Gerber, 2022).

### Therapeutic relationships

The theme ‘Therapeutic relationships’ is composed of the following five subthemes: (1) trust and therapeutic alliance, (2) the professional ethics of therapists, (3) boundary issues, (4) transference and countertransference, and (5) training for psychedelic therapists. While the first two subthemes describe more conceptual aspects of therapeutic relationships, the latter three describe contextual aspects within the therapeutic relationship and the necessary training.

While a lot of issues pertaining to the therapeutic relationship are not unique to psychedelic-assisted therapy, the intense nature of the experiences facilitated by psychedelics causes additional risks and requires clear guidelines regarding professional ethics, both during and after the treatment or trial (Azevedo et al., 2023; Rajwani, 2023; Whinkin et al., 2023) and after (Jacobs, Murphy-Beiner et al., 2023). A strong, trusting therapeutic alliance is essential, as patients often enter therapy in vulnerable states, requiring a therapist who can offer safety, empathy, and guidance throughout the process (Barnes, 1970; Ortiz et al., 2022).

Such a unique context presents higher risks of relational harm, sexual misconduct, abuse and exploitation, and raises concerns around maintaining professional boundaries, especially around therapeutic touch (Brennan & Belser, 2022; Harrison et al., 2025; Herpers et al., 2024; Jacobs, Earp, et al., 2024; McNamee et al., 2023; Poppe & Repantis, 2024). Such risks can be counterbalanced by employing two therapists of different genders for a session (Holka-Pokorska, 2023), by establishing clear guidelines about what constitutes acceptable therapeutic touch (McGuire et al., 2024; McNamee et al., 2023; Neitzke-Spruill et al., 2025; Peacock et al., 2024; Sisti, 2024; Smith & Sisti, 2021), and by increasing awareness of the power imbalance between patient and therapist (Johnson, 2021; Lee et al., 2024; Peacock et al., 2024; Repantis et al., 2024). Psychodynamic concepts such as transference and countertransference, which describe the unconscious projection of feelings, including sexualized feelings, must be navigated carefully to protect both parties from relational harm and boundary violations (Anderson et al., 2020; Jacobs, 2023; Marcus, 2023; McNamee et al., 2023; Phelps, 2017; Villiger & Trachsel, 2023). Because of the patient’s suggestible state, therapists should also be aware of the potential impact that their own beliefs and explanatory models can have on their patients, and exercise caution and epistemic humility both in regards to their understanding of the patient’s condition and to metaphysical stances such as materialism or spirituality (Borkel, Rojas-Hernández, Quintana-Hernández, & Henríquez-Hernández, 2025; Caporuscio & Fink, 2024; Cohen & Marks, 2025; Jacobs, Earp, et al., 2024; Neitzke-Spruill et al., 2024; O’Donnell et al., 2025; Poppe et al., 2025).

In addition, ensuring that therapists are properly trained in the clinical use of psychoactive substances is critical for the ethical delivery of psychedelic-assisted therapy (Barber & Dike, 2023; Belouin et al., 2022); however, some aspects of the training are still being debated, and psychedelic trainings often show inconsistent practices and standards of care (Destoop et al., 2025; Perez Rosal et al., 2024; Poppe & Repantis, 2024; Sisti, 2024). For example, it is unclear whether therapists’ prior experience with psychedelics should be encouraged or even included as part of the training and to what extend this can or should be disclosed (Anderson et al., 2020; Azevedo et al., 2023; Barber et al., 2022; Barber & Dike, 2023; Barnes, 1970; Barnett & Greer, 2021; Belouin et al., 2022; Brennan & Belser, 2022; Emmerich & Humphries, 2023; Hall, 2021; Holka-Pokorska, 2023; King & Hammond, 2021; Pilecki et al., 2021; Siegel et al., 2023; Smith & Appelbaum, 2022; Thal et al., 2022; Williams et al., 2023; Wilson-Poe et al., 2024).

### Informed consent

As a cornerstone of ethical clinical practice, the theme of ‘Informed consent’ involves both conceptual and contextual dimensions, which are captured in four subthemes: (1) disclosure, (2) decision-making capacity, (3) challenges, and (4) potential solutions.

Informed consent is rendered particularly problematic by the profound psychological effects of psychedelics and clear and thorough disclosure of potential risks and benefits is essential (Barber & Dike, 2023; Barnes, 1970; Edelsohn & Sisti, 2023; Jacobs, Earp, et al., 2024; Mathai et al., 2022; Ortiz et al., 2022; Pilecki et al., 2021). This can be rendered complex by the unpredictability of the effects, not only during the acute phase of the experience but also at the level of long-term personal transformation and belief change (Anderson et al., 2020; Barber & Dike, 2023; Egerton & Capitelli-McMahon, 2023; Emmerich & Humphries, 2023; Haeusermann & Chiong, 2024; Jacobs, 2023; Jacobs, Earp, et al., 2024; Jacobs, Murphy-Beiner, et al., 2024; Repantis et al., 2024; Villiger, 2024a, 2024b). Decision-making capacity must be assessed to ensure that participants are fully able to understand and consent to the therapy, free from coercion or undue influence. This is especially complicated during the acute experience, due to the strong subjective effects (Cheung et al., 2023; Lee et al., 2024; O′ Brien & Nutt, 2025; Otterman, 2023; Peterson et al., 2023; Poppe & Repantis, 2024; Rosenbaum, Cho, et al., 2023; Sisti et al., 2014; Villiger, 2024a, 2024b).

Other challenges include managing expectations (Elman et al., 2022), accounting for cultural differences that might create misunderstandings during the session, include provisions in case patients decide to leave the session during an altered state of consciousness, and surrogate decision-making (Azevedo et al., 2023). For these reasons, the psychedelic field might benefit from developing guidelines and requirements for ‘enhanced consent’ under psychedelics (Cohen & Marks, 2025; Destoop et al., 2025; Marks et al., 2024; Smith & Sisti, 2021; Tustison et al., 2025).

### Equity and access

The overarching theme of ‘Equity and access’ is constituted by three distinct subthemes: (1) inequity, (2) accessibility, and (3) compassionate use. The subtheme of inequity provides the central conceptual framework for this domain. The subsequent subthemes delineate the primary contexts in which inequities manifest through issues of accessibility, specific barriers to treatment, challenges in real-world access, and the particular case of compassionate use programs.

Equity and access concerns are paramount in ensuring that vulnerable populations are not ‘left behind’ in the current and future provision of psychedelic-assisted therapies. These include patients from low-income backgrounds, rural areas, or underserved racial and ethnic communities. González Romero (2023) argues that the right to health and cognitive liberty includes a right to psychedelic-assisted treatments: if psychedelic-assisted treatments become available, they should be accessible to everyone who can benefit (Destoop et al., 2025). However, equitable access is limited due to social, logistical and financial barriers (Barlett et al., 2024; Jacobs, Earp, et al., 2024; Poppe & Repantis, 2024). Most psychedelic-assisted therapy protocols are time-intensive, rendering them difficult to access for vulnerable populations with limited resources, especially when the compensation is low or nonexistent (Ortiz et al., 2022); a problem that might get worse after approval if psychedelic-assisted treatments are not approved for medical insurance reimbursement (Barnett & Greer, 2021; Noorani, 2020; Rea & Wallace, 2021).

In addition to the financial barriers, literacy levels are an issue: psychedelic experiences are often difficult to characterize, and the technical language normally used might obscure comprehension, leading to experiences of powerlessness that may hinder effective informed consent procedures and affect participants’ willingness to participate (Ortiz et al., 2022). Another issue is the lack of widespread availability, particularly in marginalized communities, creating inequities in treatment access that may lead to less safe and less effective underground use (Barber et al., 2022; Smith & Appelbaum, 2022), for which especially people of color face greater legal risks (Campbell & Williams, 2021). Another question is whether psychedelics should be made available together with other forms of mental health support in prison settings (Bayrhammer-Savel et al., 2024). Some of these problems may be remedied by representing marginalized communities at the level of patients, medical professionals, and researchers, to foster a more inclusive environment (Barber et al., 2022; Haeusermann & Chiong, 2024; Lee et al., 2024; Miceli McMillan, 2022; Ona et al., 2022; Ortiz et al., 2022; Rea & Wallace, 2021; Rosa, 2022; Whinkin et al., 2023; Williams et al., 2023). Cost-saving and access-promoting approaches include group therapy and short-acting psychedelics (Barber & Dike, 2023).

Accessibility for patients resistant to other forms of treatment should be priority. Compassionate use programs, designed to provide treatments outside of clinical trials for those without alternative options, may offer solutions but also pose questions about fairness and safety (Campbell & Williams, 2021; Greif & Šurkala, 2020; Hall, 2021).

### Research ethics

We delineated eight subthemes for research ethics. Four are core ethical concepts: (1) clinical equipoise, (2) risk and benefit analysis, (3) research integrity, and (4) social value. The other four are contextual factors specific to psychedelic research: (5) research policy, (6) vulnerable groups, (7) unique methodological challenges, and (8) the limited evidence base.

A predominant subtheme that emerged from our analysis on research ethics was the importance of research policy. This includes criticism to the Food and Drug Administration and other regulators limiting the freedom of use and research of Schedule I substances (Andreae et al., 2016) and arguments for the right to early access to promising experimental therapies for patients with mental illnesses (Black, 2023; Campbell & Williams, 2021). Research integrity is also a common concern, including researcher bias (Kious et al., 2023; Oliveira-Maia & Seybert, 2024; Tang, 2024), worries about the appropriation of publicly funded knowledge by the private sector (Ona et al., 2022), and calls for more public, independent research (Hall, 2021). Andreae et al. (2016) point out a lack of ethics guidelines on the conduct of studies with schedule I substances, while Da Costa and Sofuoglu (2023) mention the need for independent review and potential biases of research ethics committees toward experimentation with psychedelics. Many of the problems compromising the validity of psychedelic research can be attributed to its unique methodological challenges, including difficulties in trial design, expectancy effects, and unblinding (Destoop et al., 2025; Iserson, 2019; Matvey et al., 2025; Oliveira-Maia & Seybert, 2024; Scala et al., 2024).

Another key topic related to research ethics concerns harm to participants. The unpredictability of psychedelic effects and the insufficient data regarding the clinical and social values of psychedelic research (Bodnár & Kakuk, 2019; da Costa & Sofuoglu, 2023; Peterson et al., 2019) makes the risk/benefit ratio complex to calculate (Bodnár & Kakuk, 2019; O′ Brien & Nutt, 2025). Furthermore, the unpredictability of psychedelic trials and the hype around them might have an impact on the capacity to obtain informed consent for safeguarding of participants’ autonomy (Andreae et al., 2016; Bodnár & Kakuk, 2019). It is also important to respect the right of participants to withdraw from the study (Bodnár & Kakuk, 2019).

It is also crucial to consider issues pertaining to justice in research, for example, how vulnerable groups might be affected by the way psychedelic research is conducted. There is a duty to facilitate research engagement among marginalized populations (Bodnár & Kakuk, 2019), to counteract the underrepresentation of people of color (Azevedo et al., 2023; Barber & Dike, 2023; Edelsohn & Sisti, 2023; Elman et al., 2022; George et al., 2020; Simon, 2024), queer people (Bartlett et al., 2024), and women (George et al., 2020) in psychedelic research. Furthermore, psychedelic research must come to terms with its history of unethical research (Edelsohn & Sisti, 2023; Smith et al., 2022; Strauss et al., 2022; Zhang & Ho, 2017) and mitigate the risk of repeating past mistakes. For this reason, it is important to work toward equitable research practices and community-based participatory research (Williams et al., 2023).

### Special contexts

For this contextual theme, the thematic analysis resulted in four subthemes of special contexts: (1) pediatric and adolescent patients, (2) military, (3) end of life, and (4) other conditions.

Different patient populations demand special ethical considerations. For pediatric and adolescent participants, questions arise around increased vulnerability, their capacity to assent to the treatment, and their parents’ authority to give consent for them (Edelsohn & Sisti, 2023; Jeffrey et al., 2024; Sathappan & Yudkoff, 2024; Sutherland et al., 2025). This is especially relevant for conditions that have a significant adolescent population, such as Anorexia Nervosa (Hu et al., 2025; Lacroix et al., 2024; Otterman, 2023).

Patients with Alzheimer’s disease and other dementias might benefit from access to psychedelic-assisted therapy (Rudolph, 2023; Zheng et al., 2024), but these conditions magnify ethical issues relevant to broader psychedelic medicine, such as the impact of psychedelics on autonomy and consent, the impact of ‘ego dissolution’ on someone experiencing a pathology of self, the impact of psychedelics on caregiving, the effects of misleading public claims, and exaggerated hype on desperate patients (Peterson et al., 2023). Furthermore, since risk factors linked to dementia development are common among marginalized populations, there is a risk of perpetuating existing inequities (Rudolph, 2023). Similar concerns apply to disorder of consciousness patients, whose consciousness is globally impaired for an extended period of time (Cardone et al., 2024; Peterson et al., 2019). This poses questions related to surrogate consent, risk/benefit analysis, and fair participant selection (Peterson et al., 2019).

Military populations raise additional concerns (Wolfgang & Hoge, 2023), such as considerations of confidentiality (i.e. that psychedelics might predispose a patient to divulge information that they would not have intended to (Hoener et al., 2023) and personality shifts due to treatment that may make it more difficult for service members to continue actively participating in war or combat (Hoener et al., 2023. There are also concerns of weaponization and military abuse of psychedelics (Germann, 2019).

End-of-life care brings forth questions about the use of psychedelics to address existential distress, balancing relief of irremediable suffering with concerns about the safety and ethical administration of substances in terminal stages (Berens & Kim, 2022; Cornish et al., 2025; Korkmaz et al., 2024; Masse-Grenier et al., 2024; Plourde et al., 2024; Poppe & Repantis, 2024; Somers & Scheepers, 2025). One dilemma that is specific to end-of-life care regards authenticity in the case of patients changing their decision to seek medical assistance in dying following psychedelic-assisted therapy (Berens & Kim, 2022; Rosenbaum, Hales, & Buchman, 2023; Somers & Scheepers, 2025). If psychedelics get approved to treat non-psychiatric conditions, such as chronic pain, a thorough ethical assessment should address potential dilemmas before they arise (Robinson et al., 2024).

### Societal and cultural implications

The societal and cultural implications of psychedelic-assisted therapy extend beyond the clinic. In the analysis, five subthemes were salient. They entailed both conceptual and contextual themes such as (1) interactions with non-clinical uses, (2) medicalization and unique regulatory challenges, (3) cultural sensitivity and inclusivity, (4) media and the public, and (5) artificial intelligence.

It is currently unclear what effect medicalization will have on psychedelic use outside of the clinic, where psychedelic substances are often consumed either recreationally or as a form of self-improvement. While some recognize the value of psychedelics outside the clinic, others worry about the lack of safety in such contexts, which could lead to social harms or undermine therapeutic benefits (Andrews et al., 2025; Barber & Dike, 2023; Letheby & Saja, 2022; Williams et al., 2023). Inclusivity and respect for indigenous traditions are key concepts. The increasing medicalization of psychedelics raises questions about the tension between therapeutic use and the broader cultural, spiritual, and traditional practices in which these substances have historically been embedded (Andrews & Wright, 2022; Belouin et al., 2022; Celidwen et al., 2022; Cohen & Marks, 2025; Gerber et al., 2021; McGuire et al., 2024; McMillan, 2021; Miceli McMillan, 2022; Neitzke-Spruill et al., 2024; Noorani, 2020; Ona et al., 2022; Rea & Wallace, 2021; Rudolph, 2023; Whinkin et al., 2023) and privatization and financial investments (Destoop et al., 2025). This includes more specific concerns of cultural appropriation, unequitable sharing of benefits, inaccessible costs for marginalized populations, and ecological concerns (Azevedo et al., 2023; Celidwen et al., 2022; Gerber et al., 2021; Haeusermann & Chiong, 2024; Hauskeller & Schwarz, 2023; King & Hammond, 2021; Labate et al., 2022; Marcus, 2023; McMillan, 2021; Ona et al., 2022; Peacock et al., 2024; Rea & Wallace, 2021; Rosa, 2022; Schenberg & Gerber, 2022; Williams et al., 2023) as well as intellectual property rights impeding indigenous access to psychedelics (Belouin et al., 2022; Celidwen et al., 2022).

Additionally, public discourse and media portrayals of psychedelics must be carefully managed to avoid positive or negative exceptionalism about psychedelics, including misinformation, misrepresentation, hype, or stigmatization (Azevedo et al., 2023; Barber et al., 2022; Belouin et al., 2022; Beswerchij & Sisti, 2022; Campbell & Williams, 2021; Cheung et al., 2025; Cohen & Marks, 2025; Munafò et al., 2023; Noorani, 2020; O′ Brien & Nutt, 2025; Ona et al., 2022; Peterson et al., 2023; Pilecki et al., 2021; Ryan & Loo, 2017; Sandbrink et al., 2024; Sisti, 2024).

Finally, the use of new technology, such as Artificial Intelligence, in combination with psychedelic-assisted treatments is likely to become an important topic in future years and ethical implications of it should be carefully assessed (Sarris et al., 2024).

## Discussion

The results of this systematic scoping review provide the full spectrum of ethical issues in psychedelic-assisted treatments. While this scoping review described the ethical issues in the published bioethical academic literature, it is not evaluative nor prescriptive. It can therefore only serve as a steppingstone toward a nuanced discussion of the ethics of psychedelic-assisted treatments and psychedelic ethics in general (Jacobs et al., 2023), leading to their better understanding and implementation. In the following, first we discuss conclusions and open questions from the review, as well as methodological limitations. Finally, we briefly sketch needs for future research.

### The psychedelic renaissance is the heyday of psychedelic bioethics

Based on the sheer number of included articles from the last 10 years (98%), we can first conclude that the current ‘psychedelic renaissance’ is accompanied by intense academic discussion on ethical issues. This is also evident in vast number of themes and subthemes of our analysis, which depict a nuanced picture of ethical issues across clinical and research use of psychedelics. Although ongoing critical reflection is still warranted, we can be optimistic that current psychedelic researchers reflect more on inherent ethical issues as well as that there is stronger external control by Institutional Review Boards and professional organizations. From the standpoint of bioethics, this does mean that the psychedelic renaissance has surpassed early psychedelic research, potentially also enabling the current research to elude old perils.

### Relations to the four principles of medical ethics

The results of our scoping review highlight a complex landscape of challenges and considerations that directly intersect with the four principles of medical ethics: autonomy, beneficence, non-maleficence, and justice (Beauchamp & Childress, 2019). Instead of a top-down classification based on these principles, we opted for an inductive analysis of the main issues, dilemmas and solutions specific to psychedelic-assisted therapy that are addressed in the literature. As a result, the categories we have identified are not straightforwardly mapping onto these broader principles, but are often located at the intersection between two or more domains. The first two themes, ‘safety and patient well-being’ and ‘therapeutic relationship’, are strongly connected to beneficence and non-maleficence, namely, the duty to protect the patient’s best interests and avoiding harm. The central theme of informed consent relates to the principle of respect for patient autonomy, namely, the right to make informed and autonomous decisions about one’s own medical care. The theme ‘equity and access’ reflects the principle of justice, which strives for the fair and equitable distribution of healthcare resources. Additionally, we identified a category which we labeled ‘special contexts’ because it subsumes issues that become relevant when PAT is directed toward specific populations that warrant unique worries, regulations, or concerns (e.g. informed consent for adolescent patients). These issues emerge across different themes and therefore relate to all four principles of medical ethics. The five aforementioned themes are strictly related to clinical ethics. However, ethical issues related to psychedelic-assisted therapy extend far beyond the clinic. ‘Research ethics’ is not strictly clinical ethics; however, we decided to include this theme because most psychedelic treatments currently happen within clinical trials. Research ethics encompasses issues of informed consent, benevolence, non-maleficence, and justice toward the research participants; furthermore, the quality of the research has direct implications on the effectiveness, availability and cost of treatment in the clinical context, thus directly impacting the other themes we identified. Our final theme, labeled ‘social and cultural implications’, is meant to reflect on the consequences and implications of psychedelic-assisted therapy beyond the clinical or research context and for the broader society.

### Different ethics for different substances

In order to capture the ethical tensions of the whole field of psychedelic therapy, for this scoping review, we defined psychedelics broadly and included both MDMA and ketamine, which are (for a lack of a better term) ‘atypical’ psychedelics. Due to its potential for abuse and dependency, ketamine especially constitutes an outlier, which might reflect some of the specific ethical issues of ketamine therapy as discussed in detail by (Zhang & Ho, 2017) and Zhang, Harris, and Ho (2016). It seems prudent and worthwhile to distinguish the ethical issues of the therapy with entactogens such as MDMA (as e.g. pointed out by Holka-Pokorska (2023) from the ethics of classical psychedelics and theirs in turn, for example, from the ethics of the treatment with the short acting and more intense substance 5-MeO-DMT. So far, ethical issues have in general not been widely differentiated by psychedelic substance although their unique pharmacology and subjective experiences render a more differentiated and nuanced analysis of their therapeutic use and accompanying challenges.

### The need for interdisciplinary, empirical-normative research on ethical issues

Our analysis made evident that ethical issues are discussed in different research disciplines, ranging from psychiatry and psychotherapy to anthropology and philosophy. Some ethical discussions are very oriented toward the hands-on problems in research and clinical use, for example, lack of access to psychedelic-assisted therapy, while others offer fine-grained analysis of ethical issues, for example, justificatory requirements for training experiences with psychedelics. Naturally, representatives from specific disciplines in some occasions also discussed differently or only focus on particular aspects of the given matter. Here, the results indicate a need for interdisciplinary work combining normative analyses with empirical social and political sciences to lay the ground for safe and ethical implementation of psychedelic-assisted treatments.

### Limitations

As both a methodological feature and a limitation of a scoping review, the analysis has been primarily cursory. While this review does show the full spectrum of ethical issues in psychedelic-assisted therapies, it lacks depth in the analysis of specific ethical issues. Hence, the present analysis is unable to show the argumentative quality of the provided ethical issues and whether the presented ethical issues are indeed the needed priority in the field of psychedelic bioethics. One additional, reasonable explanation for that is that our analysis has been overinclusive in its depth. It included a wide range of academic articles addressing the ethics of psychedelic-assisted therapies, which in its turn were also widely defined as treatments with diverse substances such as classic psychedelics, MDMA, and dissociatives.

However, the scoping review has also been underinclusive by restricting itself to only academic, peer-reviewed literature. Ethical issues might have been more vehemently discussed in grey literature, such as policy briefs or blog posts. Indeed, the role of blogs and podcasts to provide a timely corrective to ethical missteps in the psychedelic research is not to be underestimated.

### Future research

This scoping review provides the groundwork for future research which should prioritize in-depth analysis of specific ethical issues to advance the field of psychedelic bioethics. A systematic review of reasons (as developed by Strech and Sofaer (2012) on specific ethical issues can be the next step in any given theme and subtheme of this review. Patient autonomy and informed consent, for instance, appear to be prominent issues across various ethical dimensions, as evidenced by their recurring presence in five out of the seven categories explored in this scoping review. An in-depth investigation of this topic should include an evaluation and weighing of normative reasons.

Furthermore, the integration of empirical and normative methods in research is warranted (Salloch, Schildmann, & Vollmann, 2012). Empirical research in medical ethics is crucial for grounding ethical principles in real-world data, such as surveys and interviews, and ensuring that ethical decision-making is not solely theoretical: by identifying the outcomes of ethical choices, the impact of policies, the preferences and needs of patients and clinicians and the moral intuitions of the public, it fosters a more comprehensive understanding of ethical issues, ensuring that guidelines and policies remain responsive to the complexities of healthcare environments.

## Supporting information

Caporuscio et al. supplementary material Caporuscio et al. supplementary material

## Supplementary material

The supplementary material for this article can be found at http://doi.org/10.1017/S0033291725101761.
