# Supplementary material for: Ethical issues with psychedelic-assisted treatments in psychiatry: A systematic scoping review
Source: Psychol Med. 2025 Sep 29;55:e284. doi: 10.1017/S0033291725101761 (PMC12527517; doi:10.1017/S0033291725101761)
Supplement: Caporuscio et al. supplementary material [file S0033291725101761sup001.pdf]

Supplementary Material

General Search: PCC for included studies (Population, Concept, Context): (“psychiatr*” OR synonyms OR index terms) AND (“ethic*” OR synonyms OR index terms) AND (“psychedelic*” OR synonyms OR index)

Overview of index terms (MeSH/Subject headings/Psychological Index Terms)

	PubMed/Medline	PsycArticles/Info	CINAHL
Psychiatry/Mental disorders	psychiatry/<br>mental disorders/<br>Clinical Trial/<br>psychotherapy/	Psychiatry<br>Mental Disorders<br>Clinical Trials<br>Psychotherapy	Psychiatry<br>Mental Disorders<br>Clinical Trials<br>Psychotherapy
Ethics	ethics/	Ethics	Ethics<br>Ethics theory<br>Research ethics
Psychedelics	Hallucinogens/<br>Hallucinogens [Pharmacological action]<br>Ketamine/<br>Tryptamines	Psychedelic Assisted Therapy<br>Psychedelic drugs	Hallucinogens<br>Ketamine

Example search strategy on MEDLINE via OVID and Pubmed:

OVID

(("psychiatr*".ab,ti. OR PTSD.ab,it. OR depression.ab,ti. OR “clinic*”.ab,ti. OR psychotherapy.ab,ti. OR psychiatry/ OR mental disorders/ OR Clinical Trial/ OR psychotherapy/)

AND

(“ethic*”.ab,ti. OR “moral*”.ab,ti. OR "legal*".ab,ti. OR normative.ab,ti. OR norms.ab,ti. OR (social adj issues).ab,ti. OR elsa.ab,ti. OR elsi.ab,ti. OR (social adj implications).ab,ti. OR ethics/)

AND

("psychedelic*".ab,ti. OR "entactogen*".ab,ti. OR ketamine.ab,ti. OR OR psilocybin.ab,ti. OR “magic adj mushroom*”.ab,ti. OR LSD.ab,ti. OR Lysergic Acid Diethylamide.ab,ti. OR entheogen*.ab,ti. OR 5-MeO-DMT.ab,ti. OR MDMA.ab,ti. OR ecstasy.ab,ti. OR ayahuasca.ab,ti. OR ibogaine.ab,ti. OR PCP.ab,ti OR mescaline.ab,ti. OR hallucinogens/ OR hallucinogens [pharmacological action]/ OR ketamine)

Pubmed

("psychiatr*"[Title/Abstract] OR "PTSD"[Title/Abstract] OR "depression"[Title/Abstract] OR "clinic*"[Title/Abstract] OR "psychotherapy"[Title/Abstract] OR (psychiatry OR mental disorders OR Clinical Trial OR psychotherapy[MeSH Terms])) AND (("ethic*"[Title/Abstract] OR

"moral*"[Title/Abstract] OR "legal*"[Title/Abstract] OR normative[Title/Abstract] OR "norms"[Title/Abstract] OR "social issues"[Title/Abstract] OR "social implications"[Title/Abstract]) OR (ethics[MeSH Terms])) AND (("psychedelic*"[Title/Abstract] OR "entactogen*"[Title/Abstract] OR ketamine[Title/Abstract] OR psilocybin[Title/Abstract] OR "magic mushroom*"[Title/Abstract] OR "LSD"[Title/Abstract] OR "Lysergic Acid Diethylamide"[Title/Abstract] OR "entheogen*"[Title/Abstract] OR "5-MeO-DMT"[Title/Abstract] OR "MDMA"[Title/Abstract] OR "ecstasy"[Title/Abstract] OR "ayahuasca"[Title/Abstract] OR "ibogaine"[Title/Abstract] OR "PCP"[Title/Abstract] OR "mescaline"[Title/Abstract]) OR (hallucinogens OR hallucinogens [pharmacological action] OR ketamine[MeSH Terms]))
